# Transitional Care Interventions for Patients with Heart Failure: An Integrative Review

**DOI:** 10.3390/ijerph17082925

**Published:** 2020-04-23

**Authors:** Hai Mai Ba, Youn-Jung Son, Kyounghoon Lee, Bo-Hwan Kim

**Affiliations:** 1 Department of Nursing, Gachon University Graduate School, Incheon 21936, Korea; maibahai211@gmail.com; 2 Red Cross College of Nursing, Chung-Ang University, Seoul 06974, Korea; yjson@cau.ac.kr; 3 College of Medicine, Division of Cardiology, Gachon University, Incheon 21565, Korea; cardioman@gilhospital.com; 4 Cardiovascular Research Institute, Gachon University, Incheon 21565, Korea; 5 College of Nursing, Gachon University, Incheon 21936, Korea

**Keywords:** heart failure, integrative review, transitional care intervention

## Abstract

Heart failure (HF) is a life-limiting illness and presents as a gradual functional decline with intermittent episodes of acute deterioration and some recovery. In addition, HF often occurs in conjunction with other chronic diseases, resulting in complex comorbidities. Hospital readmissions for HF, including emergency department (ED) visits, are considered preventable. Majority of the patients with HF are often discharged early in the recovery period with inadequate self-care instructions. To address these issues, transitional care interventions have been implemented with the common objective of reducing the rate of hospital readmission, including ED visits. However, there is a lack of evidence regarding the benefits and adverse effects of transitional care interventions on clinical outcomes and patient-related outcomes of patients with HF. This integrative review aims to identify the components of transitional care interventions and the effectiveness of these interventions in improving health outcomes of patients with HF. Five databases were searched from January 2000 to December 2019, and 25 articles were included.

## 1. Introduction

Heart failure (HF) is a life-threatening syndrome in which the cardiac pump does not sufficiently maintain the blood flow to meet the body’s needs for oxygen and blood [1]. HF syndrome constitutes a major global health problem, affecting at least 26 million people worldwide [2,3]. In addition, the prevalence of HF will dramatically increase with an aging population. In the United States, there are currently 5.7 million cases of HF, with the projected annual incidence expected to exceed 8 million by 2030 [4]. Likewise, the prevalence of patients with HF in Korea was estimated to be 1.53% in 2013 and is expected to increase 2.2-fold to 3.4% by 2040. In other words, over 1.7 million Koreans are estimated to be affected by HF by 2040 [5].

HF contributes to substantial morbidity, high mortality, frequent readmissions, and emergency department (ED) visits because of progressive HF pathogenesis, poor self-management, and emergent signs and symptoms owing to increased hemodynamic overload [1]. Consequently, these vicious cycles negatively affect patients’ quality of life (QoL) [6] and health-related QoL (HRQoL) [7] as well as increase the burden of healthcare cost on patients and their families [8].

Despite advancements in medical procedures and treatments, HF management remains a challenge to healthcare providers. Of the various methods to manage HF, transitional care intervention is the most innovative program to improve the continuity of care for patients with HF from admission to after hospital discharge [9,10]. A transitional care intervention based on transfer from hospital to home was designed, evaluated, and implemented by Mary Naylor at the University of Pennsylvania in Philadelphia to improve the outcomes of chronic older patients as well as to reduce the costs of healthcare [11]. According to the American Heart Association, transitional care interventions for HF should span the care continuum [10]. Specifically, the most important core features of transitional care are comprehensive in-hospital planning, postdischarge follow-up, and ongoing support via telephone or home visits for chronically ill, high-risk, older patients hospitalized for medical services [10,11].

Until now, there have been some systematic reviews or meta-analyses of randomized controlled trials (RCTs) reporting on transitional care interventions [9,12,13]. There have also been reports on the evidenced effectiveness of transitional care interventions based on non-RCT study designs. Integrative reviews combine data of both experimental and non-experimental studies to fully understand the phenomenon being analyzed. In addition, integrative reviews are the best comprehensive methodological approach to evaluate theoretical and empirical literature with a range of purposes, such as analysis of methodological problems related to a topic [14]. Therefore, the specific objectives of this integrative review were to analyze the components of transitional care intervention and examine the effectiveness of these interventions in improving clinical and patient-related outcomes of HF.

## 2. Materials and Methods

### 2.1. Study Design

This integrative literature review studies and summarizes previous research by drawing conclusions from individual studies believed to address the relevant topics. The integrative review methodology described by Whittemore and Knafl [14] was used. This review includes five stages: problem identification, literature search, data evaluation, data analysis, and presentation.

### 2.2. Problem Identification

The central question of this integrative review is “What are the outcomes/effectiveness of transitional care intervention/transitional care programs for HF?” The characteristics evaluated included completeness of the intervention; nature of the intervention (educational alone or including multiple interventions); who was the target population; and what were the outcomes (clinical outcomes: all-cause mortality rates, readmission rates, average number of ED visits per patient, length of stay (LOS) in index care, index care costs, and follow-up costs; patient-related outcomes: QoL, HRQoL, satisfaction, and quality of transition).

### 2.3. Literature Search

We performed a search for relevant articles in the databases of PubMed, Cumulative Index of Nursing and Allied Health Literature, Web of Science, EMBASE, and COHRANE. The keywords used to search were as follows: (1) “transitional care intervention” OR “transitional care program” OR “transition of care” OR “transition of care model” OR “postdischarge follow up” and (2) “HF patients.” Articles included in this integrative review met the following criteria: (1) focus on adult HF patients, (2) concern for the effectiveness of transitional care interventions or transitional care programs, (3) clinical and patient-related outcomes mentioned in stage 1 as dependent variables, (4) patients transiting from the hospital to home, (5) accessibility of the full text of articles in detail, (6) published in English, and (7) published between January 2000 and December 2019. Non-original research articles, study protocols, development of instrument, and studies that did not clearly describe the transitional care procedure or the process of intervention were excluded. Two authors (H.M.B. and B.-H.K.) independently screened the studies according to the criteria and discussed the screening results. Finally, 25 papers met the criteria and were included in this review.

### 2.4. Data Evaluation

The quality of studies with different designs was assessed using the Mixed Methods Appraisal Tool (MMAT; 2018 version), which is a critical tool designed for the appraisal stage of systematic mixed methods studies, including integrative reviews [15]. MMAT enables the appraisal of the methodological quality based on the following five categories of studies: qualitative research, RCTs, non-randomized studies, quantitative descriptive studies, and mixed methods studies. Computing the score from the grading of each item is discouraged. Instead of computing the score, a direction to write a more detailed evaluation of each item to better assess the quality of the included studies is used. Therefore, excluding studies with low methodological quality is usually discouraged.

The 25 included studies were evaluated using MMAT. The two screening questions were applied to all reviewed studies regardless of the design. Afterward, the appropriate criteria were selected and answered according to each study design. Two authors (H.M.B. and B.-H.K.) independently evaluated the studies according to the criteria and discussed the results.

All 25 studies satisfied the two screening questions. Of the eight RCTs, seven satisfied all quality appraisal criteria and one showed a higher dropout rate in the control group than in the intervention group, resulting in mortality bias [16]. Of the 15 quantitative non-randomized studies, 13 met all quality appraisal criteria, one showed a rate of complete outcome data of <80% [17], and another lacked inclusion and exclusion criteria for participant selection [18]. Of the two quantitative descriptive studies, one satisfied all criteria [19] and the other lacked criteria for the risk of non-response bias; moreover, the authors did not balance for any potential confounding factors such as patient age, level of education, or duration of HF [20].

### 2.5. Data Analysis

**Figure 1:**
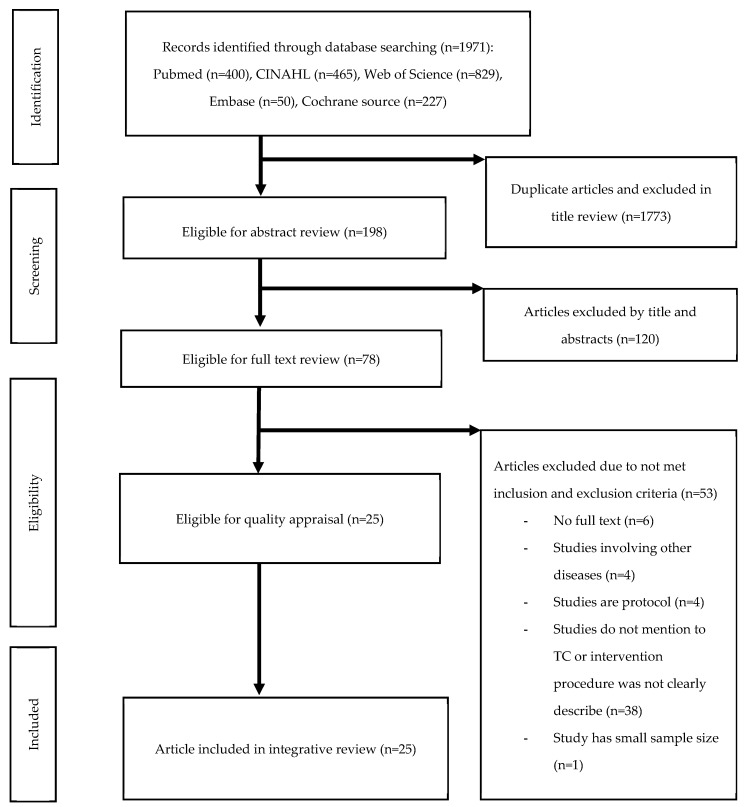
Preferred reporting items for systematic reviews and meta-Analyses (PRISMA) flow diagram outlining the literature search and study selection.

We fully read and analyzed each of the 25 selected studies independently. We met regularly for review, data reduction, and data extraction to complete the integrative review. The characteristics and results of the studies are summarized in the order of year in Table 1. The three sections in Table 1 are as follows: (1) general study information (author (year), country, study design, sample size (male %), type of HF, age, racial ethnicity, and HF severity); (2) major information regarding transitional care interventions (intervention, major intervention provider, transition time/place, and intervention duration); and (3) major outcomes (clinical and patient-related outcomes). The effectiveness of transitional care interventions for patients with HF was analyzed in two domains: clinical outcomes (all-cause or HF readmission rate, ED visit, mortality rate, LOS, and care costs) and patient-related outcomes (QoL, HRQoL, satisfaction, and quality of transition). In addition, the intervention components were analyzed according to previous transitional care models and are summarized in Table 2. These include (1) predischarge and (2) postdischarge interventions. Figure 1 presents a flow diagram of all review stages.

## 3. Results

### 3.1. Characteristics of the Included Studies

This review included 25 studies regarding transitional care interventions for patients with HF that met our search criteria. In total, 17 of the 25 studies were conducted in North America [17,18,19,20,21,22,24,25,26,27,29,31,34,37,38,39,40], three in Europe [23,30,35], three in China [16,28,32], and two in Iran [33,36]. Thirteen of the 25 studies did not report racial ethnicity [16,20,21,23,24,26,28,30,32,33,35,36,38]. The sample size ranged from 38 [24] to 3462 [19], and the average patient age was between 65 [36] and 82 years [27,30]. The percentage of males ranged from 35% [34] to 97% [37], but three studies [20,26,32] did not report sex.

### 3.2. Type and Severity of HF

Subjects presented with various HF types: two studies reported on end-stage HF [28,32], three on chronic HF [16,23,33], two on congestive HF [21,37], one on decompensated HF [29], and the remaining 17 on HF [17,18,19,20,22,24,25,26,27,30,31,34,35,36,38,39,40]. To identify HF severity, we considered the New York Heart Association (NYHA) class and left ventricle ejection fraction (LVEF). Only 10 of the 25 studies reported NYHA class or LVEF [16,19,21,22,24,28,29,34,36,40]. One study reported B-type natriuretic peptide level >200 ng/mL [25], and two studies reported HF with reduced or preserved ejection fraction [17,18]. The remaining 12 studies [20,23,26,27,30,31,32,33,35,37,38,39] did not report the participants’ severity of HF.

### 3.3. Methodology of Transitional Care Interventions

All studies conducted transitional care interventions for patients with HF. Regarding research design, there were 10 RCTs [16,21,22,24,28,29,32,33,36,38], two quasi-experiments [23,40], two pre- and post-tests [17,26], six cohort studies [18,19,27,34,35,37], and five prospective studies [20,25,30,31,39]. Although it was necessary to establish controls to verify the effects of transitional care interventions, in six of the 25 studies [17,18,19,20,26,39], there was no comparative control group set up to verify the effectiveness of transitional care interventions. In 15 of the 25 studies, the primary intervention providers were nurses [16,20,21,22,23,24,25,26,28,29,30,32,33,37,38], including advanced practical nurses, clinical nurse specialists, research nurses, nurse practitioners, cardiac nurses, registered nurses, nurse case managers, registered nurse case managers, and liaison nurses. A multidisciplinary team served as the primary intervention provider in five studies [17,31,35,36,39]. In the remaining five studies [18,19,27,34,40], the major intervention providers were pharmacists or pharmacy students.

Most transitional care intervention supported patients with HF when they directly transitioned from hospital to home. In two studies, patients were transitioned or referred to a transition clinic [17] or structured multidisciplinary outpatient clinic for old and frail postdischarge patients hospitalized for HF [30].

There were different intervention durations, ranging from 2 weeks [18,21,24,31] to 15 months [34]. Five of the 25 studies frequently conducted transitional care interventions for 3 months [20,22,25,28,36]. Of the 25 studies, four were conducted for 2 weeks [18,21,24,31], four for 4 weeks [17,30,37,40], and three for 6 months [29,33,38].

### 3.4. Outcomes of Transitional Care Intervention

We attempted to separate the outcomes of the included studies into two categories: clinical and patient-related outcomes. Clinical outcomes included all-cause [16,17,18,19,20,21,22,23,25,26,27,28,29,30,31,32,35,36,37,38,39,40] and HF-related [19,30,34,40] readmission or rehospitalization, ED or urgent care visit [21,32,37,38,39], outpatient visit to a physician [36], mortality or death rate [16,29,38,39], event-free survival [16], and LOS [16,23,25]. Patient-related outcomes included self-reported or interviewed measurements such as QoL [21,22,28,29,32,38], HRQoL [16,17,21,24,33], satisfaction [22,23,28], medication adherence [24,36], self-efficacy for HF self-care, self-care, symptom intensity, functional status [16], quality-adjusted life years [32,38], and discharge preparedness or quality of transition [38]. Two of the 25 studies [24,33] did not measure clinical outcomes and 13 [18,19,20,25,26,27,30,31,34,35,37,39,40] did not measure patient-related outcomes.

### 3.5. Effects of Transitional Care Intervention

The highest frequency of the measured clinical outcomes was for readmission or rehospitalization. Twelve of the 22 studies that measured readmission/rehospitalization showed statistically significant reduction in the rate of readmission/rehospitalization [16,18,22,25,27,28,30,31,32,36,37,40], although the 10 remaining studies did not [17,19,20,21,23,26,29,35,38,39]. The second highest frequency of clinical outcomes was for ED visit [21,32,37,38,39] reported in five studies, followed by cost [22,25,32,37] and HF readmission [19,30,34,40] each reported in four studies. Generally, although transitional care interventions tended to reduce cost, the trend was not statistically significant [25,32,37]. Only one study showed a significant reduction of cost [22]. Moreover, transitional care interventions significantly reduced the rate of ED visits [21,32] in two of the five studies as well as the rate of HF readmission [30,40] in two of the four studies. Regarding the aspects of mortality [16,29,39] and LOS [16,23,25], transitional care interventions significantly reduced mortality and LOS in one [16] of the three studies.

The highest frequency of the measured patient-related outcomes was for QoL [21,22,28,29,32,38]. In four of the six studies, QoL was significantly betted in the experimental group than in the control group [22,28,29,38]. However, five studies reported significantly deteriorated HRQoL [16,17,21,24,33]. Satisfaction with transitional care interventions was significantly greater in the experimental group than in the control group [22,28]. Medication adherence was significantly reduced in one [24] of the two studies [24,36]. Quality-adjusted life years were not significantly changed in two studies [32,38], but discharge preparedness or quality of transition were significantly better in one study [38].

### 3.6. Components of Transitional Care Interventions

The components of transitional care interventions in this study were temporally categorized into two durations: pre- and postdischarge interventions.

#### 3.6.1. Predischarge Interventions

A total of 21 of the 25 studies offered predischarge interventions in their transitional care programs [16,17,18,19,21,22,23,25,26,27,28,29,32,33,34,35,36,37,38,39,40]. In five studies, early assessment or daily monitoring was implemented after hospital admission [18,21,22,27,39]. In two studies, an appointment schedule was planned before discharge to review the required care [16,28]. Fourteen studies [17,21,23,25,26,27,28,29,34,35,37,38,40] showed that predischarge health education and counseling were an important component of transitional care intervention that includes discharge planning, health education, discharge counseling, and patient-centered discharge instructions. Medication reconciliation upon admission, on discharge, and during hospitalization was described in seven studies [19,21,27,34,35,39,40], and this was also a critical component for improving medication safety in patients with HF. Only one study [35] focused on other components of transitional care intervention, including emotional support by nurses and notification to the general practitioner. Nurses sent a message to the general practitioner regarding patient information before discharge, and community nurses also sent notifications if the patients received assistance from community nursing services.

#### 3.6.2. Postdischarge Interventions

A total of 24 of the 25 studies [16,17,18,19,20,21,22,23,24,25,26,27,28,29,30,31,32,33,34,35,36,37,38,39] emphasized on postdischarge interventions as a main component of transitional care. The patterns of intervention differed across studies, although their nature focused on four main characteristics, namely telephone support, follow-up telephone calls, nurse home visits, and other additional care for outpatients. Three studies [22,25,26] provided telephone support by an advanced practical nurse available 7 days per week and included education and consulting. Ten studies [16,21,22,23,25,28,30,31,32,38] offered home visits by HF nurses to monitor and manage signs and symptoms after discharge as well as to deliver patient education. One study [32] combined both nurse and volunteer social visits for patients with HF after discharge. Another study [18] involved support from social workers to provide the necessary support and to ensure that the transition services were delivered with comprehensive intervention. Twenty-two studies [16,17,18,19,20,22,23,24,25,26,27,28,29,30,32,33,34,35,36,37,38,39] reported that early follow-up after discharge and follow-up telephone calls were an advanced intervention in transitional care for patients with HF. This was shown to support the postdischarge education materials and to identify patient’s understanding of medication changes and management of HF symptoms since discharge via telemonitoring. Additional care interventions for outpatients after discharge included supportive care for self-management [21], connections between the hospital and community nurses and patients [21], appointments for transition clinic visits to assess risk factors [17], and physical and treatment evaluations of patients [20,38].

## 4. Discussion

In this integrative review, we confirmed the variety and complexity of transitional care interventions that have been proposed. First, we found that transitional care interventions were temporally analyzed over two durations, pre- and postdischarge. Of the 25 studies, 20 [16,17,18,19,21,22,23,25,26,27,28,29,32,33,34,35,36,37,38,39] offered transitional care interventions both before and after discharge. Most transitional care interventions began immediately after hospital admission [16,17,18,19,21,22,23,25,26,27,28,29,32,33,34,35,36,37,38,39,40] and continued for varying periods after hospital discharge [16,17,18,19,20,21,22,23,24,25,26,27,28,29,30,31,32,33,34,35,36,37,38,39]. The primary predischarge interventions included discharge planning, patient health education, and counseling [17,21,23,25,26,27,28,29,34,35,37,38,40]. Moreover, many interventions involved appointment scheduling before discharge [16,28] as well as explaining or introducing telephone calls or home visits after discharge [32,36]. According to the European Society of Cardiology/American Heart Association guidelines, a multidisciplinary team approach and effective systems of care coordination with special attention to care transitions highlight the importance of preventing readmission or mortality of patients with HF after hospital discharge [41,42]. Transitional care interventions support safe, smooth, and efficient quality transitions and are mainly focused on transition from hospital to home [10]. Unfortunately, despite the need to accurately identify patients’ problems and to develop tailored transitional care interventions when patients with HF are admitted to a hospital, early assessment of such patients for transition from hospital to home was presented in only five of the 25 studies [18,21,22,27,39]. Many care management interventions for HF have traditionally focused on patients with chronic HF during their outpatient phase [43]. Contrary to this, transitional care interventions managed by a multidisciplinary team have focused on transitions, particularly between the acute and postdischarge phases [17,31,35,36,39]. Transitional care interventions have strengths including early assessment of patients’ needs and expected health problems in the home setting before beginning discharge planning for the admission duration. Regarding the assessment of patients’ and their families’ knowledge and understanding of HF, health providers should begin discharge planning for patients with HF based on their baseline understanding and knowledge of HF that can be corrected before hospital discharge [10].

Second, we analyzed the outcomes of transitional care intervention, namely clinical and patient-related outcomes. The most frequent clinical outcome measure was readmission after hospital discharge. We identified that transitional care intervention significantly reduced the rate of readmission in approximately 55% (12 of 22 studies) of studies [16,18,22,25,27,28,30,31,32,36,37,40] in this review. Similar to the present review, some reviews and meta-analyses of patients with congestive HF have reported significantly reduced risks of readmission with transitional care interventions [12,44,45]. Moreover, the most frequent patient-related outcome measures were QoL [21,22,28,29,32,38] and HRQoL [16,17,21,24,33] after hospital discharge. We identified that transitional care intervention significantly improved QoL or HRQoL in more than 90% of studies in this review [16,17,21,22,24,28,29,33,38]. Likewise, another integrative review has reported that transitional care interventions could improve QoL of patients with HF [45]. Transition from hospital to home is a vulnerable time for patients and their families. Therefore, to improve the safety of transition, healthcare providers must measure the quality of the transition [46]. Surprisingly, only one [38] of the 25 studies in this review reported discharge preparedness and quality of transition for patients with HF during intervention periods. Although the results of that single, large medical-center study [38] generally support the association between quality of transition (3-Item Care Transitions Measure score) and readmission, they should be interpreted with caution. Of note, the authors recommend that this association should be validated at a large scale, at a hospital level, in future studies [47]. Specially, we identified that transitional care intervention was mainly delivered by nurses in this review, and 12 nurse-led transitional care intervention studies reported significantly improved clinical and patient-related outcomes [16,21,22,24,25,28,29,30,32,33,37,38]. According to a systematic review and meta-analysis, nursing activities such as home visits, case management, and disease management clinics where physicians and cardiac nurses work together with a multidisciplinary HF management team showed significantly decreased all-cause readmission and death [9]. Moreover, in some interventions for heart diseases, the effects of nurse-led transitional care programs appeared excellent [16,48]. We confirmed that nurses are the most important healthcare providers of transitional care for patients with HF.

Third, in two large-scale RCTs [29,38], patient-related outcomes but not clinical outcomes were statistically meaningful. This may be because of some specific problems. For instance, in one study performed at 10 hospitals selected via randomization in Canada [38], the eligibility criteria were broad, and patients were included regardless of age, left ventricular function, comorbidities, or prognosis. In another study performed in the United States [29], the better effectiveness after transition-heart failure (BEAT-HF) intervention via telemonitoring did not show significant effects on all-cause readmission within the first 30 or 180 days. These results are related to daily weight changes and physiological signals associated with aggravated symptoms in patients with HF. Moreover, in that study, adequate warnings could not be provided to patients with decompensated HF that recurrence of HF is imminent. Therefore, when conducting a large-sample RCT of transitional care interventions, participant selection criteria should be strictly homogeneous between control and intervention groups, the severity of HF should be assessed based on NYHA class or LVEF, and disease-related conditions should be recorded. Because these factors can affect the risk of readmission or ED visit, they should be taken into account. When providing transitional care, high intensity of interventions and continuous contact of patients with HF with healthcare providers might be crucial to reduce the risk of readmission [44]. Consequently, through meticulous research, transitional care interventions should be tailored according to the severity of disease to achieve the best clinical outcomes in patients with HF.

Finally, HF is a global health concern mostly affecting older people, and research in this setting must consider social, economic, and cultural contexts [49]. Nevertheless, the studies reviewed herein highlight that over 68% of publications on transitional care interventions for HF are concentrated in North America. In addition, 40% of studies in North America and Europe did not consider racial ethnicity, which could affect cultural contexts. In these countries, the importance of transitional care intervention has been demonstrated in terms of effectively preventing patients’ readmission or ED visits and reducing their mortality. However, in Asia, including Korea, there is still little evidence regarding the effectiveness of transitional care interventions for patients with HF. Thus, research in this area is urgent. Similar to our finding, a previous review has also pointed out the importance of research conducted in various countries [50].

### Limitations

We searched and included only English publications. Therefore, this review synthesizes the best available evidence published in English alone. Moreover, we did not search for all gray literature databases, such as dissertation databases and non-peer reviewed articles, or internet-based search engines (e.g., Google and Google Scholar). Therefore, all relevant research may not have been included in this integrative review.

## 5. Conclusions

Our findings highlight that transitional care interventions should be developed at the time of patient admission. Transitional care interventions should include early assessment of disease knowledge, with an understanding of what the patients with HF need for self-care at home in order to enhance safe transition from hospital to home. Specifically, before discharge from hospital, the quality of transition should be monitored to confirm that the patient is ready for self-care at home. In addition, we recommend extensive research in Asia, including Korea, to increase the effectiveness of transitional care interventions for patients with HF. We must remember that the role of nurses is very important to realize a good overall multidisciplinary team approach.

## Figures and Tables

**Table 1:** Descriptive summary of transitional care intervention studies for patients with HF (*n* = 25).

Author (Year) Country	Study Design	Sample (Number (Male %), Type of HF, Age, Racial Ethnicity, HF Severity)	Contents of Intervention (Intervention, Major Intervention Provider, Transition Time/Place, Intervention Duration)	Outcomes (Significance)	
				Clinical Outcomes	Patient-Related Outcomes
Harrison, et al. (2002) [21] Canada	RCT (Exp. vs. Con.)	N = 192 (55%)	Transitional care intervention	Hospital readmissions (−)	HRQoL (+++)
		Congestive HF	Nurses		
		75 years	Hospital to home	All-cause emergency room visits (+)	QoL (−)
		N/A			
		NYHA III/IV 77%	Until 2 weeks after hospital discharge		
Naylor et al. (2004) [22] UnUSA	RCT (Exp. vs. Con.)	N = 239 (43%)	Transitional care intervention (APN-directed discharge planning and home follow-up)	First rehospitalization period or death at 52 weeks (+)	QoL (+)
		HF			
		76 years			
			APN		
		African American 36%	Hospital to home		Satisfaction (+++)
		LVEF (~45%–20%) ~69%–72%	3 months	Cost (+)	
Williams et al. (2010) [23] United Kingdom	Quasi-experiment (Exp. vs. Con.)	N = 97 (52%)	Transitional care service	Readmissions at 30 days (−)	Satisfaction (non-statistics, positive feedback)
		CHF			
		~71–78 years	CNS		
		N/A	Hospital to home	LOS (−)	
		N/A	18 weeks		
Barnason et al. (2010) [24] USA	RCT (Exp. vs. Con.)	N = 38 (65%)	Hospital transition intervention	N/A	Medication adherence (+++)
		HF	Research nurse		
		77 years	Hospital to home		Self-efficacy for HF self-care (+++)
		N/A			
		NYHA III 55%	2~3 weeks		HRQoL (++)
Stauffer et al. (2011) [25] USA	Prospective study (Exp. vs. Con.)	N = 1025 (47%)	Transitional care program	30 day all-cause readmission rate (+)	N/A
		HF	APN	LOS (−)	
		~79–81 years			
		White ~78%–84%	Hospital to home	60 day direct cost from admission (−)	
		BNP level > 200 ng/mL	3 months	Budget impact analysis (non-statistics, reduced hospital financial)	
Simpson (2014) [26] USA	Pre-and post-test (Exp. only)	N = 263 (N/A)	Nurse-implemented transitional care	30 day readmission rate (non-statistics, decreased rate)	N/A
		HF	NP		
		N/A			
		N/A	Hospital to home		
		N/A	5 months		
Yu et al. (2015) [16] China	RCT (Exp. vs. Con.)	N = 178 (45%)	Cardiac nurse-implemented transitional care	Event-free survival (−)	Self-care (maintenance, management, confidence, and knowledge) (+) HRQoL (++)
		CHF	Cardiac nurse		
		79 years	Hospital to home	All-cause hospital readmission (+)	
			9 months		
		N/A		9 month mortality (+)	
		NYHA II/III ~97%–98%		LOS (+)	
Truong et al. (2015) [27] USA	Cohort study (Exp. vs. Con.)	N = 632 (~49%–61%)	Continuum of Care Network (CCN) program	30 day all-cause hospital readmissions (++)	N/A
		HF	Resident pharmacist		
		~68–82 years			
		White ~59%–62%	Hospital to home	Compliance with HF-1 at a single community hospital (++)	
		N/A	From admission to home after discharge		
Wong et al. (2016) [28] China	RCT (Exp. vs. Con.)	N = 84 (52%)	Transitional Care Palliative (TCP)-ESHF program	Readmissions at 12 weeks (++)	Symptom intensity (+)
					Functional status (+)
					QoL (+)
		ESHF	NCM		
					Satisfaction with care (+++)
		78 years	Hospital to home		
		N/A			
		NYHA III/IV 86%~93%	12 weeks		
Ong et al. (2016) [29] USA	RCT (Exp. vs. Con.)	N = 1437 (54%)	Better Effectiveness After Transition–Heart	180 day all-cause readmission (−)	QoL (+)
		Decompensated HF	Failure (BEAT-HF)		
		73 year	Nurses		
		White ~54%–55%	Hospital to home	30 day all-cause readmission (−)	
		African American ~22%–			
		23%	180 days	180 day mortality (−)	
		NYHA III ~64%–66%			
O’Connor et al. (2016) [20] USA	Prospective study (Exp. only)	N = 818 (N/A)	Telehealth program using the Transitional Care Model	All-cause 30 day readmission rate (non-statistics, reduced readmission)	N/A
		HF	RN and telehealth liaisons nurses		
		N/A			
		N/A	Hospital to home		
		N/A	Mean 63~94 days		
Whitaker-Brown et al. (2017) [17] USA	Pre-and post-test (Exp. only)	N = 50 (42%)	4-week pilot transition-to-care program	30 day hospital readmission (non-statistics, two participants were readmitted)	HRQoL (+)
		HF	Multidisciplinary team		
		70 years	Hospital to outpatient setting (Transition clinic)		
		Caucasian 83%			
		HFrEF (Severe <20%)			
		HFrEF (Severe <20%) 11% /HFpEF (Mild 40%–55%) 19%	4 weeks		
Pacho et al. (2017) [30] Spain	Prospective study (Exp. vs. Con.)	N = 518 (43%)	APN-directed discharge planning and home follow-up protocol	All-cause 30 day readmission (+++)	N/A
		HF			
		82 years	APN		
		N/A	Hospital to STOP-HE-clinic	HF-related 30 day readmission (+++)	
		N/A	30 days		
Miller et al. (2017) [31] USA	Prospective study (Exp. vs. Con.)	N = 462 (49%)	Multidisciplinary post-acute transitional care (MDTC) program	All-cause readmission rates (+++)	N/A
		HF			
		81 years	Multidisciplinary team	Visit number during the first 2 weeks (++)	
		Caucasian 79.5%	Hospital to home (connected by Hospital home care agency)		
		N/A	2 weeks		
Wong et al. (2017) [32] China	RCT (Exp. vs. Con.)	N = 84 (N/A)	Transitional Home-based Palliative End-stage Heart Failure (THPESHF) program	Readmission at 84 days (+++)	QoL (−)
		ESHF			
		76 years		ER visit at 84 days (+)	QALY (non-statistics, 0.0012 at 28 days/0.0077 at 84 days)
		N/A	NCM	Hospital stay at 84 days (+++)	
			Hospital to home	Cost (non-statistics, cost-effectiveness probability)	
		N/A	1 year		
Rezapour-Nasrabad (2018) [33] Iran	RCT (Exp. vs. Con.)	N = 168 (63%)	Transitional care intervention	N/A	HRQoL (+)
		CHF	Liaison nurses		
		>65 years (30%)	Hospital to home		
		N/A			
		N/A	6 months		
Moye et al. (2018) [34] USA	Cohort study (Exp. vs. Con.)	N = 177 (35%)	Pharmacy team-led intervention program	The number of days that elapsed after discharge to the first readmission (+)	N/A
		HF	Pharmacist	HF-related readmission (−)	
		71 year	Hospital to home		
		African American 92%	15 months		
		LVEF (<40%) ~41%–48%			
Garnier et al. (2018) [35] Switzerland	Cohort study (Exp. vs. Con.)	N = 1872 (~53%–54%)	Multimodal care transition plan	The fraction of days spent for readmissions (−)	N/A
		HF	Multidisciplinary team	The rate of readmission (−)	
		~76–78 years	Hospital to home	Decreasing the fraction of days spent for 30 day readmission compared to non-completers (++)	
		N/A			
		N/A	13 months	Decreasing PARE compared with non-completers (++)	
				The rate of PARE decreased ~8.7%–9.9%, reaching the adjusted expected range given by SQLape^®^ (7.7%–9.1%)	
Shekarriz-Foumani et al. (2018) [36] Iran	RCT (Exp. vs. Con.)	N = 120 (~65%–73%)	Education and Follow-up after Discharge	Readmission rate (+)	Medication compliance (−)
		HF			
		~65–66 years	(FAD) program	Outpatient visits to physician (−)	
		N/A	Multidisciplinary team		
		NYHA III/IV ~42%–45%	Hospital to home		
			3 months		
Reese et al. (2019) [37] USA	Cohort study (Exp. vs. Con.)	N = 1092 (97%)	The Coordinated-Transitional Care (C-TraC) program	Readmission (+)	N/A
		Congestive HF		30 day ED or UC visits (−)	
		~74–75 years	RN-CM	Cost (non-statistics, TCI helps decrease total cost)	
		White ~90%–91%	Hospital to home		
		N/A	4 weeks		
Van Spall et al. (2019) [38] Canada	RCT (Exp. vs. Con.)	N = 2494 (50%)	Patient-Centered Care Transitions in HF (PACT-HF) service	All-cause readmission at 30 days (−), 3 months (−)	Discharge preparedness at 6 weeks (+++)
		HF			
		78 years			
			NCM	ED visit at 30 days (−), 3 months (−)	Quality of transition at 6 weeks (+)
		N/A	Hospital to home		
		N/A	6 months	Death at 3 months (−)	QoL at discharge (+++), 6weeks (+), and 6 months (+)
					QALY (−)
Murphy et al. (2019) [39] USA	Prospective study (Exp. only)	N = 100 (58%) in HF	Cardiac Transitions of Care Pilot Program	30 day readmission rates (−)	N/A
		HF	Multidisciplinary team (physicians, pharmacists, nurse practitioners, dietitians)	72 h ED visit rates (−)	
		68 years	Hospital to home	30 day mortality rate (−)	
		Caucasian 69%			
		N/A	5 weeks (Inpatient 1 week and outpatient 4 weeks)		
Plakogiannis et al. (2019) [18] USA	Retrospective cohort study (Exp. only)	N = 131 (57%)	Transdisciplinary HF care transition team (HFCTT) intervention with pharmacy student–driven postdischarge phone calls	Readmission: at 30 days (++) and 90 days (++)	N/A
		HF	Pharmacy student (with Multidisciplinary team)		
		72 years			
		White 71%	Hospital to home		
		HFrEF 48%	Different duration by each patient ~14–60 days		
Wood et al. (2019) [19] USA	Retrospective cohort study (Exp. only)	N = 3462 (56%)	Transitions of Care (TOC) Pharmacist Services	30 day all-cause readmission (−)	N/A
		HF			
		72 years	Pharmacists and an HF nurse educator		
		White 95% of n = 2347	Hospital to home		
		LVEF (≤40%) 26%	From admission to ~48–72 h after discharge	30 day HF readmissions (−)	
Neu et al. (2020) [40] USA	Quasi-experiment (Exp. vs. Con.)	N = 663 (52%)	Pharmacy-led HF transition of care (TOC)	HF 30 day hospital readmission rate (+)	N/A
		HF			
		~66–69 years	Pharmacist		
		White 40%, Black 57%	Hospital to home		
		LVEF (≤40%) ~51%–54%	30 days		

Statistical significance: + *P* < 0.05; ++ *P* < 0.01; +++ *P* < 0.001; − *P* > 0.05. AMI, acute myocardial infarction; APN, advanced practice nurses; BHCS, Baylor Health Care System; BMCG, Baylor Medical Center Garland; BNP, B -type natriuretic peptide; CAN, care assessment needs; CHF, chronic heart failure; CNS, clinical nurse specialist; CON, control; COPD, chronic obstructive pulmonary disease; C-TraC, coordinated-transitional care; ED, emergency department; EF, ejection fraction; ESHF, end-stage heart failure; HF, heart failure; HFCTT, HF Care Transitions Teams; HFrEF, heart failure reduced ejection fraction; HRQoL, health-related quality of life; LOS, length of stay; LVEF, left ventricle ejection fraction; NP, nurse practitioner; NCM, nurse case manager; NYHA, New York Heart Association; PT, physical therapist; PARE, potentially avoidable readmission; QALYs, quality-adjusted life years; QoL, quality of life; RN-CM, registered nurse case manager; RCT, randomized controlled trial; STOP-HF-Clinic, STructured multidisciplinary outpatient clinic for Old and frail Postdischarge patients hospitalized for HF; TCI, transitional care intervention; UC, urgent care; N/A, not available.

**Table 2:** Transitional care intervention components pre- and postdischarge.

Author (Year)	Transitional Care Intervention Components	
	Predischarge Intervention	Postdischarge Intervention
Harrison et al. (2002) [21]	1. Early assessment after hospital admission; 2. Medication reconciliation; 3. Discharge planning patient education	1. Supportive care for self-management through education or home visit; 2. Links between hospital and home nurses and patients; 3. Balance of care between the patient and family and professional providers
Naylor et al. (2004) [22]	1. Early assessment after hospital admission	1. Telephone support; 2. Nurse home visits
Williams et al. (2010) [23]	1. Discharge planning patient education	1. Follow-up and home visit
Barnason et al. (2010) [24]		1. Early follow-up after discharge; 2. Follow-up telephone call
Stauffer et al. (2011) [25]	1. Screening for eligibility within hospital admission; 2. Discharge planning	1. Early follow-up after discharge; 2. Telephone support; 3. Nurse home visits
Simpson (2014) [26]	1. Education	1. Postdischarge telephone contact
Yu et al. (2015) [16]	1. Appointment schedule before discharge; 2. Discharge planning	1. Home visits; 2. Follow-up telephone call
Truong et al. (2015) [27]	1. Admission medication review; 2. Daily monitoring; 3. Discharge medication review; 4. Discharge counseling	1. Early follow-up after discharge
Wong et al. (2015) [28]	1. Appointment schedule before discharge; 2. Discharge planning	1. Home visit together; 2. Telephone follow-up
Ong et al. (2016) [29]	1. Predischarge health education	1. Follow-up telephone call
O’Connor et al. (2016) [20]		1. Telemonitoring (personal goal setting, self-monitoring, management of symptoms, and reporting changes to their physician or care team)
Whitaker-Brown et al. (2015) [17]	1. Discharge planning	1. Appointment for transition clinic visit (risk assessment, physical assessment, and evaluation); 2. Medication reconciliation; 3. Early follow-up telephone call; 4. Providing information related to rehabilitation, home care, hospice, and/or palliative care
Pacho et al. (2017) [30]	N/A	1. Early postdischarge visit; 2. HF nurse education to patient and caregiver; 3. Treatment titration; 4. Intravenous medication; 5. Early follow-up via e-notification
Miller et al. (2017) [31]	N/A	1. Education and consulting; 2. Early home visit
Wong et al. (2017) [32]	1. Hospital visit before discharge to introduce the program	1. Nurse home visit; 2. Nurse telephone call; 3. Volunteer social visit
Rezapour-Nasrabad (2018) [33]	1. Nursing care support	1. Follow-up telephone call
Moye et al. (2018) [34]	1. Medication prescript and manage; 2. Standard-of-care HF education program; 3. Medication reconciliation; 4. Discharge planning	1. Postdischarge appointment; 2. Follow-up phone calls by pharmacy team
Garnier et al. (2018) [35]	1. Targeted therapeutic education; 2. Caregiver therapeutic education; 3. Medication reconciliation at admission and discharge; 4. Set up of an appointment with the GP; 5. Notification of the GP; 6. Community nurse notification; 7. Patient-centered discharge instructions	1. Follow-up telephone call; 2. Telephone support
Shekarriz-Foumani et al. (2018) [36]	1. Screening for eligibility within hospital admission; 2. Collecting demographic and disease information; 3. Explaining questions to be interviewed on telephone calls	1. Educating the patients and their guardians immediately after discharge; 2. Follow-up telephone call
Reese et al. (2019) [37]	1. Discharge planning patient education	1. Telephone follow-up
Van Spall et al. (2019) [38]	1. Nurse-led self-care education; 2. A structured hospital discharge summary	1. Family physician follow-up; 2. Postdischarge nurse-led home visits and heart function clinic care (includes telephone assessment)
Murphy et al. (2019) [39]	1. Admission medication review. 2. Daily monitoring 3. Discharge medication review	1. Discharge counseling by telephone; 2. Postdischarge follow-up
Plakogiannis et al. (2019) [18]	1. Early assessment after hospital admission	1. The social worker provided the patient and the caregiver with the necessary support for a smooth transition into the community; 2. Telephone call by pharmacy student (reviewed the medications, HF symptoms, and performed a detailed medication reconciliation and counseling)
Wood et al. (2019) [19]	1. Inpatient medication reconciliation; 2. Medication history review	1. A follow-up phone call
Neu et al. (2020) [40]	1. Admission medication reconciliation; 2. Discharge medication reconciliation; 3. Patient or caregiver counseling with a focus on HF medications through verbal and written education materials	
